# Optimization of Mechanical and Dynamic Properties of Tread Rubber Using Fumed Silica and Hydration Processing

**DOI:** 10.3390/polym17060714

**Published:** 2025-03-07

**Authors:** Qingchen Chu, Xiaolong Tian, Huiguang Bian, Chuansheng Wang

**Affiliations:** 1 School of Mechatronics Engineering, Qingdao University of Science and Technology, Qingdao 266061, China; 2022030013@mails.qust.edu.cn (Q.C.); tianxiaolong@qust.edu.cn (X.T.); bianhuiguang@qust.edu.cn (H.B.); 2 National Engineering Laboratory of Advanced Tire Equipment and Key Materials, Qingdao University of Science and Technology, Qingdao 266061, China

**Keywords:** fumed silica, tread rubber, natural rubber, dry-mixed rubber compound, hydration process

## Abstract

Fumed silica, a nanomaterial with a high specific surface area, excellent chemical stability, and electrical insulation, serves as an effective filler for rubber compounding. Compared to traditional carbon black, silica (SiO_2_), the main component of fumed silica, improves the hardness and tear resistance of tread rubber, making it a viable substitute in some formulations. However, silica-filled compounds generally exhibit lower tensile properties and abrasion resistance than carbon black. Fumed silica, with its higher structural integrity, provides additional reinforcement points within natural rubber matrices, enhancing tensile strength and abrasion resistance. Studies demonstrate that replacing carbon black with an equivalent amount of fumed silica as the primary filler significantly improves tread rubber’s hardness (by 20%) and 300% tensile modulus (by 14%) while also reducing rolling resistance and enhancing wet skid performance. Fumed silica’s large specific surface area and low density (10–15% of conventional silica) make it challenging to use directly as a tread rubber filler due to dust formation and prolonged mixing times. This study developed a process combining fumed silica with deionized water, followed by drying and ball milling. This treatment reduces the material’s volume, forming a cohesive gel that, upon processing, minimizes dust and significantly decreases mixing time and difficulty. The interaction between the hydroxyl (–OH) groups on the surface of fumed silica and water molecules likely results in hydrated silica. This interaction enhances surface polarity and forms a hydration layer, improving the hydrophilicity and dispersion of fumed silica in rubber matrices. This reduces the shear modulus difference (ΔG′) between low and high strain, maintaining a consistent elastic modulus over a wide strain range. Such stability enables rubber to perform better under dynamic loads or in complex working conditions. The experimental results demonstrate that the hydration–ball milling process enhances the tensile strength of vulcanizates, improves the dispersion of fumed silica in rubber, strengthens the filler network, boosts dynamic performance, and enhances the wet skid resistance of tread rubber.

## 1. Introduction

Fumed silica is a nanoparticle material characterized by its high specific surface area and numerous reactive hydroxyl groups on the surface. Through interactions with rubber matrices such as natural rubber (NR) and styrene-butadiene rubber (SBR), fumed silica establishes significant physical and chemical bonds that enhance the strength and elastic modulus of rubber [1,2,3].

The specific surface area of fumed silica ranges from 200 to 500 m^2^/g, with particle sizes typically between 10 and 40 nm. Smaller particle sizes lead to larger specific surface areas, increasing contact with rubber matrices and providing enhanced reinforcement [4]. When dispersed in rubber, fumed silica boosts the material’s elastic modulus, improving resistance to deformation. The rigid nanoparticles dissipate stresses during deformation through inter-particle friction, reducing direct matrix damage. Furthermore, the nanoparticles endure a portion of the frictional forces during wear, improving the abrasion resistance and longevity of rubber products [5,6,7].

In addition to reinforcement, the interfacial interactions between fumed silica and rubber molecules generate physical constraints. These interactions form numerous rubber–filler interfaces, restricting the mobility of rubber chains and enhancing tensile strength and wear resistance [8]. The high modulus of fumed silica particles helps distribute and absorb external stresses, reducing localized stress concentrations and preventing premature failure of the rubber material [9,10].

At higher filler loadings, silica particles form a three-dimensional (3D) network through physical contacts and van der Waals forces [11,12,13]. This structure increases material rigidity, modulates particle spacing under dynamic stress, and reduces internal stress concentrations, delaying fatigue failure and improving tear resistance and dimensional stability. Additionally, the internal dissipation of mechanical energy during dynamic deformation enhances damping properties and reduces rolling resistance [14,15].

Under specific temperature and pressure conditions, fumed silica filler may induce micro-phase separation within the rubber matrix [16,17]. This creates additional crosslinking points or interaction zones, strengthening the rubber matrix and increasing its adhesion and viscosity [18,19,20,21].

The use of nanosilica as a filler to enhance the properties of rubber materials has been widely studied. However, reinforcement with a single filler has limited the application of rubber composites, as it may not meet the requirements of specific environments. As a result, nanocomposite fillers have attracted attention from researchers, such as silica/carbon black, silica/clay, and silica/carbon nanotube composites. These hybrid fillers, when added to rubber, make the material suitable for more specialized applications, thereby broadening its use across various industries.

Nanocomposites refer to materials in which at least one phase has a one-dimensional size ranging from 1 to 100 nm. The unique properties of nanocomposites, such as their excellent overall performance and specific functionalities, arise from the small size effect of the dispersed phase, their large surface area, as well as interface effects and quantum effects, including macroscopic quantum tunneling.

However, simple mechanical blending alone does not significantly enhance the performance of rubber materials. As a result, the preparation of composite materials has become a focal point in rubber filler research [22].

Kong et al. [23] used multi-walled carbon nanotubes and fumed silica as dual-component fillers. Prior to blending, the components were pretreated to create chemical bonds between MWCNTs and SiO_2_. This study was the first to modify MWCNTs with methyl diethoxy silane (MDES) and SiO_2_ with vinyltriethoxysilane, followed by a platinum-catalyzed hydrosilylation reaction to create the CNTs-b-FSiO_2_ binary nanofiller. The results indicated that the CNTs-b-FSiO_2_ composite significantly improved the mechanical properties of sulfur rubber, such as tensile strength, tear strength, and Young’s modulus. This enhancement is primarily due to the strong chemical bonding between CNTs and FSiO_2_, which leads to a strong interaction, while the TEVS molecules on FSiO_2_ improve the interfacial interaction between the composite filler and the SR matrix.

Sahar Ziraki et al. [24] reinforced high-temperature vulcanized silicone rubber with polypropylene fibers and nanosilica. The results showed that the composite effectively reduced the compression set of the silicone rubber while slightly increasing the rubber’s glass transition temperature (Tg). Cao et al. [25] prepared a composite material by filling graphene oxide (RGO) and KH550- and Si69-modified nanosilica into natural rubber. The composite effectively enhanced the energy storage modulus and wear resistance of the natural rubber while reducing the loss factor and elongation at break. Adding 1% by weight of this composite to car tires increased the tire’s wear resistance by 44.5%, reduced rolling resistance by 5.1%, and improved wet skid resistance by 14.6%.

D’Arienzo et al. [26] used polyhedral oligomeric silsesquioxane (POSS) to composite with modified nanosilica, creating POSS@SiO_2_ nanocomposites. First, they surface-modified nanosilica with 3-(trimethoxysilyl)propyl methacrylate (TMMS) and grafted varying amounts of POSS onto the surface of nanosilica. Similarly to silane coupling agents, the organic groups of the cage-like POSS structure tightly bound the nanosilica to styrene-butadiene rubber (SBR). During vulcanization, the curing agent dicumyl peroxide (DCP) prevented the acrylate structure in POSS from being degraded, ensuring that the silica nanoparticles remained tightly linked within the POSS nanocage. This improved the interaction between the filler and the rubber matrix. The authors incorporated the POSS@SiO_2_ nanocomposite into an SBR matrix, resulting in the POSS@SiO_2_/SBR composite. Their research showed that the presence of POSS significantly improved the modulus of the rubber composite under different strain conditions.

### 1.1. Surface Modification and Interaction Mechanisms

The surface of fumed silica contains numerous silanol (Si–OH) groups, which can chemically bond with rubber molecules to form strong interfacial interactions. To enhance compatibility with hydrophobic rubber matrices, silane coupling agents such as sulfur-containing silane (TESPT) are often used. These agents possess dual functional groups: one reacts with silica’s hydroxyl groups to form covalent bonds, while the other reacts with the unsaturated double bonds in rubber during vulcanization, creating a robust chemical network [27,28,29,30,31].

Fumed silica has high surface energy and a strong tendency to agglomerate within the rubber matrix, leading to poor dispersion [32]. Silane coupling agents modify silica’s surface, reducing its surface energy and minimizing agglomeration. This ensures uniform filler distribution and enhances mechanical and dynamic properties such as tensile strength, tear resistance, and fatigue resistance [33,34].

### 1.2. Industrial Challenges and Solutions

Fumed silica’s extremely high specific surface area and nanometric particle size (10–40 nm) result in significant dusting during handling, negatively affecting industrial production environments [35,36]. Direct use as a filler requires prolonged mixing times to achieve uniform dispersion in rubber. To address this, this study introduces a pretreatment process: fumed silica is mixed with deionized water, dried, and ball-milled. This process transforms the material into a viscous gel, reduces its volume, and eliminates dust formation [16,37,38].

Upon contact with water, hydroxyl groups on the silica surface interact with water molecules to form hydrated silica. This hydration enhances surface polarity, improves dispersion within rubber matrices, and reduces the shear modulus difference (ΔG′) between low- and high-strain states [39]. Consequently, rubber maintains a stable elastic modulus over a wide range of deformations, improving its performance under dynamic loads and complex conditions [40].

### 1.3. Experimental Results

This study demonstrates that the hydration–ball milling process significantly reduces production challenges without compromising the mechanical properties of the rubber compound. The vulcanizates showed slight improvements in wet skid performance due to hydration, as water molecules formed hydrogen bonds with silica’s hydroxyl groups, increasing surface polarity and hydrophilicity.

### 1.4. Applications and Benefits

Fumed silica modified with silane coupling agents serves as an effective reinforcing filler, improving the mechanical strength, thermal stability, and hydrophobicity of rubber composites. Its high electrical resistivity (approximately 10^12^ Ω·m) makes it an ideal insulating material for rubber compounds [41,42]. Additionally, the inclusion of fumed silica in formulations for industrial rubber products, such as fluororubber, hydrogenated nitrile butadiene rubber (HNBR), and BR/SBR/IR blends, enhances their Payne effect, damping properties, and dynamic performance, striking a balance between rolling resistance and wet skid resistance [43,44,45].

**Figure 1:**
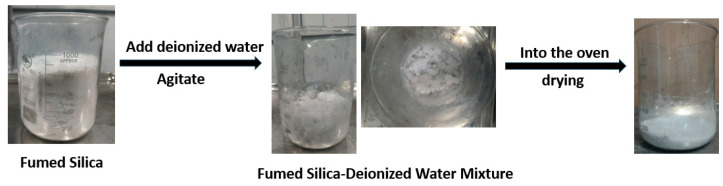
Pretreatment process of fumed silica.

By incorporating hydrated and surface-modified fumed silica, this study provides a practical solution to the challenges of industrial rubber production, ensuring enhanced performance and environmental safety. The pretreatment process of fumed silica is shown in Figure 1

## 2. Experimental Methods

### 2.1. Experimental Process and Testing

**Figure 2:**
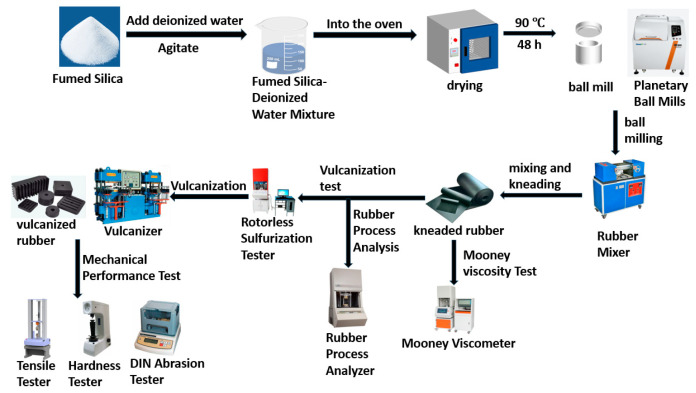
Experimental processes and testing procedures.

The experimental workflow is illustrated in Figure 2.

(1) Preparation of Fumed Silica Samples

A beaker containing fumed silica was slowly filled with deionized water at three times the weight of the silica while continuously stirring with a glass rod. The mixture was then placed in an oven set to 90 °C for 48 h. Afterward, the material was removed, and its moisture content was tested. Once the moisture content was below 0.5%, the material was subjected to planetary ball milling for 3 h. The hydrated fumed silica obtained from this process was labeled Sample A and used as the primary filler in the third experimental group of rubber compounds.

(2) Preparation of Rubber Compounds

The specified quantities of smoked sheet rubber, fillers (e.g., pyrolytic carbon black or ball-milled Sample A), zinc oxide, and other rubber additives were weighed according to the formulation.

A Banbury mixer was preheated to an initial temperature of 100 °C and set to a rotational speed of 90 rpm.

Natural rubber was added and masticated for 60 s before introducing the filler (pyrolytic carbon black or Sample A), zinc oxide, and other additives.

The mixing continued until the material temperature reached 145 °C, where it was maintained for 90 s before discharging the compound.

The compound was then processed on an open mill, during which the vulcanization system (including HDOT20, accelerator DZ, and CTP) was added. The rubber sheet was folded and rolled three times to achieve uniform dispersion, forming the rubber compound.

(3) Preparation of Vulcanized Rubber

The rubber compounds were left to rest for over 8 h before testing. Samples weighing at least 6 g were taken for curing characteristic testing using an MDR-C rotorless rheometer. The vulcanization temperature was set to 150 °C, and key parameters such as optimum cure time were recorded

For vulcanization:

The vulcanization press was set to 150 °C with a pressure of 10 MPa.

Thin products were cured for 1.3 times the T90, while thick products were cured for 2 times the T90.

The rubber compounds were placed in the appropriate molds for vulcanization, and the rolling direction of the compounds was marked.

(4) Performance Characterization and Testing

Hardness: Tested following GB/T 531.1-2008 [46] (ISO 48-5:2018) [47].

Tensile and Tear Properties: Measured in accordance with GB/T 528-2009 [48] and GB/T 529-2008 [49] (ISO 37:2024 [50] and ISO 34-1:2022 [51]).

Dynamic Mechanical Properties: Evaluated under the following conditions:

Dynamic stress: 60 N, dynamic strain: 0.25%.

Static stress: 70 N, static strain: 5%.

Temperature range: −65 °C to 65 °C, heating rate: 2 °C/min.

Frequency: 10 Hz.

### 2.2. Experimental Formulations and Design

#### 2.2.1. Control Group Formulation

The control Group formulation is shown in Table 1.

#### 2.2.2. Experimental Group 1

In this group, 1165MP silica was used to replace N326 carbon black on an equivalent weight basis. To ensure compatibility between SiO₂ and rubber, an appropriate amount of silane coupling agent Si69 was added. The formulation for Experimental Group 1 is shown in Table 2.

#### 2.2.3. Experimental Group 2

In this group, fumed silica was used to replace N326 carbon black on an equivalent weight basis. Silane coupling agent Si69 was also added to improve compatibility between SiO₂ and rubber. The formulation for Experimental Group 2 is shown in Table 3.

#### 2.2.4. Experimental Group 3

In this group, hydrated fumed silica (Sample A) was prepared by mixing fumed silica with deionized water in a 1:3 mass ratio. The mixture was oven-dried at 90 °C for 48 h, and the dried sample was ball-milled for 3 h using a planetary ball mill. The resulting hydrated and ball-milled fumed silica (Sample A) was used to replace N326 carbon black on an equivalent weight basis. Silane coupling agent Si69 was added to ensure compatibility between SiO₂ and rubber. The formulation for Experimental Group 3 is shown in Table 4.

### 2.3. Instruments and Equipment

The different pieces of equipment used in this study are listed in Table 5.

## 3. Experimental Results and Analysis

### 3.1. Mooney Viscosity and Vulcanization Characteristics

#### 3.1.1. Mixing and Vulcanization Characteristics of the Compounds

The vulcanization characteristics and Mooney viscosity of the four rubber compounds with different fillers are shown in Table 6.

#### 3.1.2. Mooney Viscosity

Mooney viscosity reflects the flowability and viscosity of rubber compounds during processing. Lower Mooney viscosity typically indicates better processability, while higher values suggest increased filler content or higher compound viscosity. The samples had the following properties:

The N326 carbon black compound had the lowest Mooney viscosity, indicating the best processability.

The 1165MP silica compound showed a slight increase in viscosity compared to N326.

The increase in Mooney viscosity indicates that the compounds with fumed silica and hydrated fumed silica have higher filler contents and a greater interaction between the filler and rubber matrix. This leads to a more viscous compound, resulting in increased processing difficulty. The higher specific surface area of fumed silica contributes to this increased viscosity, making the rubber mixture more resistant to flow compared to the N326 carbon black-filled compound.

#### 3.1.3. Vulcanization Times (t_10_ and t_90_)

t_10_ represents the scorch time (the time for the vulcanization reaction to start), while t_90_ represents the time to reach 90% of complete vulcanization.

Shorter t_10_ indicates a faster vulcanization initiation, and shorter t_90_ signifies a quicker overall vulcanization process.

Comparing the samples:

The N326 carbon black compound had the longest t_10_ (4.01 min) and t_90_ (28.36 min), indicating the slowest vulcanization process.

The 1165MP silica compound reduced t_10_ by 34% and t_90_ by 53% compared to N326.

The fumed silica compound further reduced t_10_ by 53% and t_90_ by 56%.

The Sample A compound (hydrated fumed silica) showed reductions of 46% in t_10_ and 40% in t_90_ relative to N326.

The faster vulcanization observed in the silica and fumed silica compounds can be attributed to the improved surface characteristics and reactivity of the silica fillers. Specifically, hydrated fumed silica interacts more effectively with the rubber matrix, promoting faster crosslinking during vulcanization. This accelerated vulcanization process leads to significantly shorter scorch and vulcanization times (t_10_ and t_90_) compared to the N326 carbon black compound, which can enhance production efficiency.

However, while this faster curing process improves efficiency, it also increases the risk of scorch, which is an undesirable premature crosslinking that can occur during processing. The reduced scorch time may limit the processing window, requiring more precise control over vulcanization parameters such as temperature and time. Industrial processes must adjust to this accelerated curing rate to avoid defects, such as scorching or premature curing, which could negatively affect the final rubber product’s performance and quality. Effective control strategies, including optimized temperature regulation and vulcanization monitoring, are essential to mitigate the risk of scorch and ensure that the desired material properties are achieved.

#### 3.1.4. Torque Characteristics (ML, MH, and MH-ML)

M_L_ (minimum torque) represents the initial viscosity of the compound at the processing temperature.

M_H_ (maximum torque) reflects the crosslink density of the compound after vulcanization, with higher values indicating greater hardness and strength of the vulcanized rubber.

M_H_-M_L_ represents the torque increment during vulcanization, indicating the degree of crosslinking.

From the experimental data:

The fumed silica and Sample A compounds had significantly higher M_H_, M_L_, and M_H_-M_L_ values compared to the N326 compound.

The higher maximum torque (MH) and the greater torque increment (MH-ML) in the fumed silica and Sample A compounds suggest that these fillers promote a higher degree of crosslinking. This results in a stronger and harder vulcanized rubber. The enhanced crosslink density can be attributed to the superior reinforcement ability of fumed silica, which forms a denser filler network within the rubber matrix. The increased interaction between the filler and the rubber molecules contributes to greater mechanical strength and durability, particularly in high-performance applications.

### 3.2. Analysis of Vulcanized Rubber Performance

#### 3.2.1. Hardness

**Figure 3:**
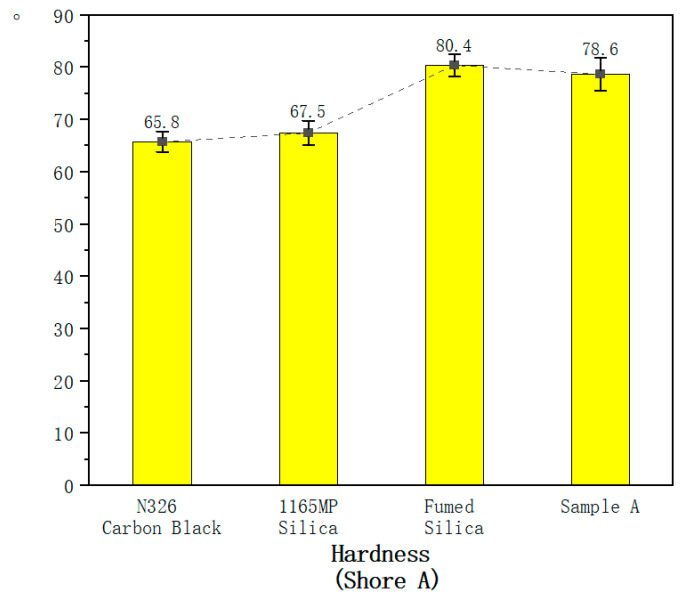
Hardness of the hybrid rubber.

**Figure 4:**
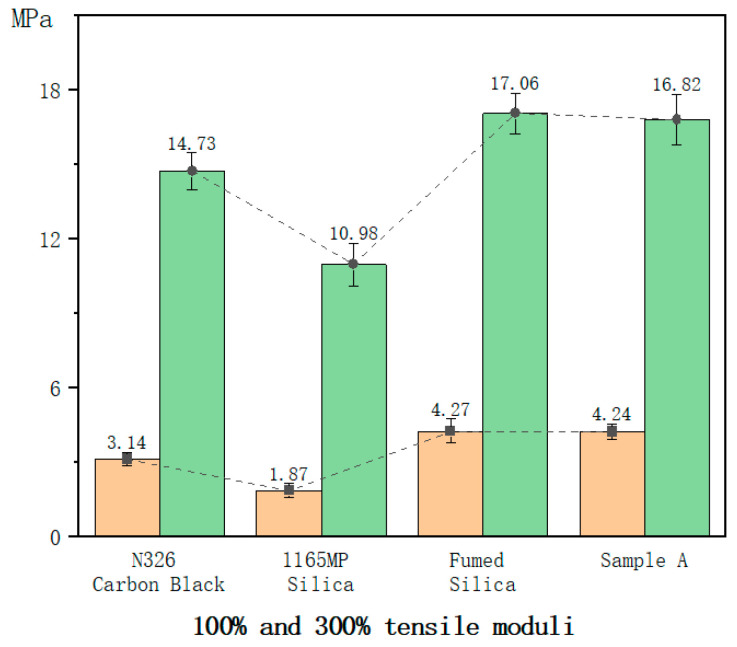
The 100% and 300% constant tensile stress of the hybrid rubber.

**Figure 5:**
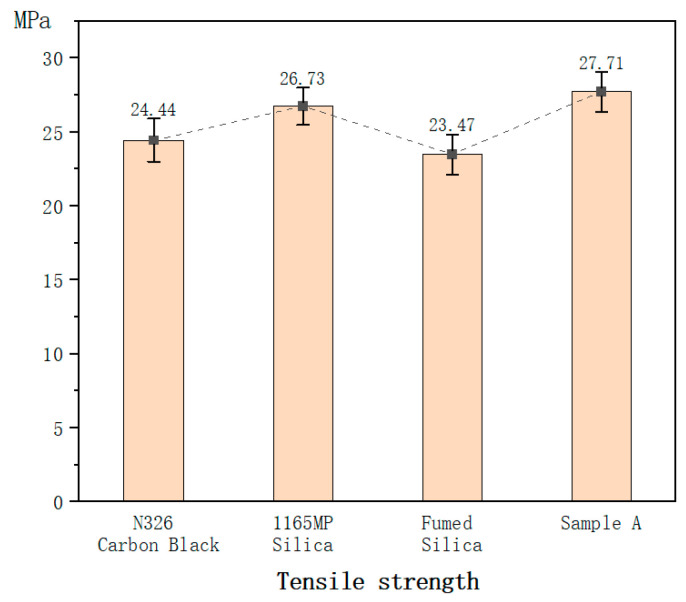
Tensile strength of the hybrid rubber.

**Figure 6:**
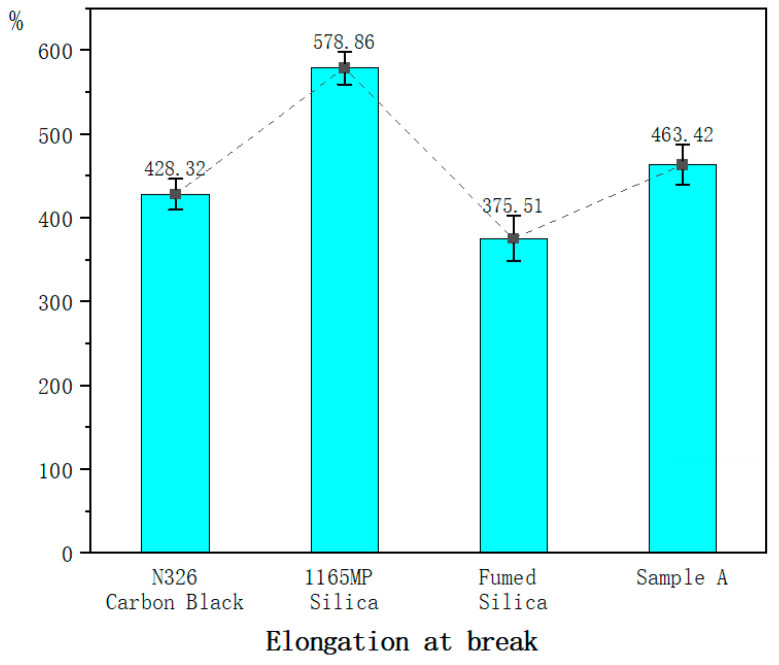
Elongation at break of the hybrid rubber.

**Figure 7:**
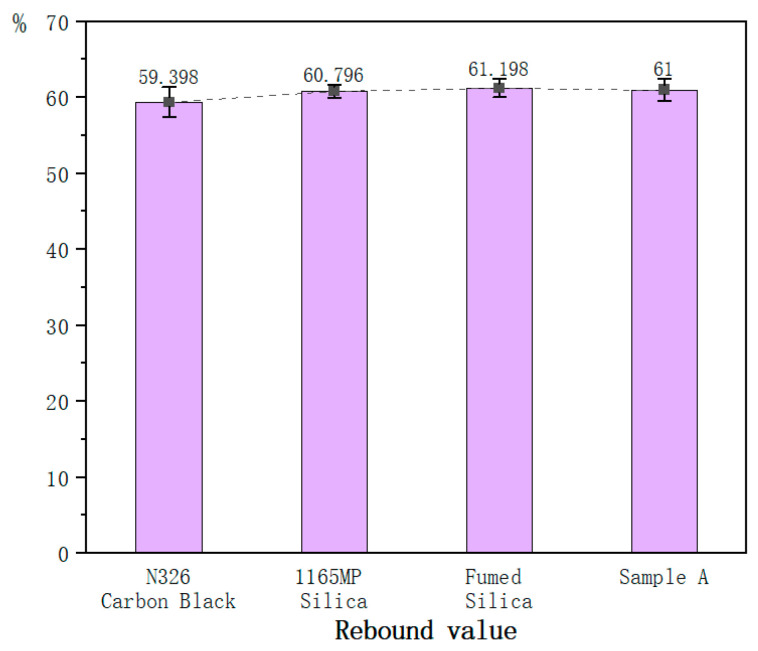
Resilience of the hybrid rubber.

**Figure 8:**
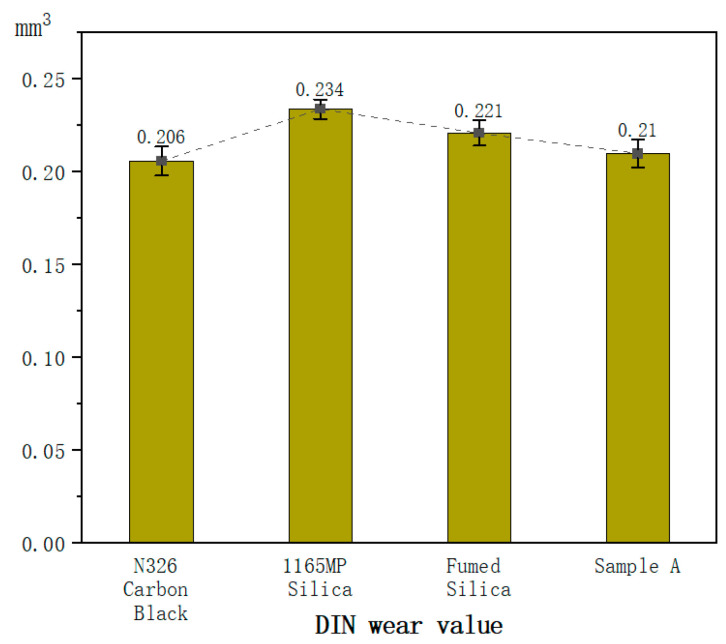
Abrasion resistance of the hybrid rubber.

For the mechanical property tests of the vulcanized rubber, each test is conducted using six specimens, all from the same batch of compounded rubber and the same vulcanization mold. The values in Table 7 represent the average of six tests, while the error bars in Figure 3, Figure 4, Figure 5, Figure 6, Figure 7 and Figure 8 are based on the standard deviation of these six tests.

According to Table 7, the hardness of vulcanized rubber containing fumed silica and Sample A (hydrated fumed silica) increased by approximately 20% compared to N326 carbon black and precipitated silica. This indicates that fumed silica significantly enhances the hardness of vulcanized rubber, consistent with its higher crosslink density observed in the vulcanization characteristics.

Both N326 carbon black and precipitated silica show lower hardness compared to fumed silica and Sample A. Carbon black, while commonly used for reinforcing rubber, typically leads to lower crosslink density compared to fumed silica, resulting in lower hardness. Precipitated silica also provides reinforcement, but its surface area and dispersion capabilities are not as pronounced as those of fumed silica, leading to a comparatively lower hardness.

Fumed silica significantly increases the hardness of vulcanized rubber. The observed 20% increase in hardness is primarily due to the higher crosslink density provided by fumed silica during the vulcanization process. Fumed silica, with its small particle size and large surface area, facilitates stronger interactions between the silica and rubber chains. This leads to an increased number of crosslinking points, making the rubber matrix more rigid, which translates to higher hardness.

Sample A (hydrated fumed silica) also showed a significant increase in hardness, although slightly lower than fumed silica. The hydration treatment introduces a thin layer of water on the silica surface, reducing the interaction strength between the silica and rubber matrix. This effect slightly mitigates the increased rigidity from fumed silica, but the hydration treatment still results in a higher crosslink density than precipitated silica and carbon black, maintaining a relatively high hardness.

#### 3.2.2. Tensile Modulus

1165MP silica compounds exhibited reductions of 32% and 34%, respectively, compared to N326 carbon black, indicating weaker reinforcement in these properties.

Fumed silica, however, increased the 100% and 300% tensile moduli by 27% and 14%, respectively, compared to carbon black, demonstrating superior reinforcement capability.

Sample A showed similar 100% and 300% tensile modulus values to fumed silica, suggesting that the hydration treatment had little impact on fumed silica’s intrinsic reinforcing ability.

The lower 100% and 300% tensile moduli of the 1165MP silica compound relative to N326 carbon black suggest that this precipitated silica provides weaker reinforcement in terms of modulus, likely due to its relatively lower surface activity and weaker filler–rubber interactions. In contrast, the fumed silica and Sample A compounds exhibited higher moduli, indicating a stronger reinforcing effect. The ability of fumed silica to enhance the modulus is attributed to its extensive hydrogen bonding and three-dimensional network, which restricts rubber chain mobility. The hydration treatment in Sample A had a minimal impact on modulus, suggesting that it does not significantly alter the intrinsic reinforcing characteristics of fumed silica.

#### 3.2.3. Tensile Strength

Precipitated silica improved the tensile strength by 9% compared to carbon black.

In contrast, fumed silica showed a slight reduction in tensile strength relative to carbon black.

However, Sample A improved the tensile strength by 16% compared to fumed silica, indicating that the hydration treatment slightly enhances its reinforcing effect on tensile strength.

The moderate increase in tensile strength observed in precipitated silica compared to carbon black indicates that silica fillers can contribute to improved tensile properties. However, the slight reduction in tensile strength in the fumed silica compound suggests that, despite its strong reinforcement effects, the higher crosslink density may lead to brittleness, reducing the ability of the rubber matrix to sustain higher tensile loads. The hydration treatment in Sample A slightly mitigates this effect, as evidenced by its higher tensile strength compared to the unmodified fumed silica compound. This suggests that hydration processing may enhance the filler dispersion and improve rubber–filler interfacial bonding, leading to improved load-bearing capacity.

#### 3.2.4. Elongation at Break

Precipitated silica increased the elongation at break by 26% compared to carbon black.

Fumed silica reduced the elongation at break by 14%, highlighting its tendency to restrict rubber chain mobility.

Sample A mitigated this issue, increasing elongation at break by 19% compared to fumed silica. This demonstrates that the hydration treatment alleviates the reduction in elongation associated with fumed silica, improving ductility.

Precipitated silica increased the elongation at break by 26% compared to carbon black. This is likely due to its higher surface area and better dispersion properties. The enhanced dispersion of precipitated silica within the rubber matrix helps reduce internal stress concentrations, improving the material’s ability to deform and increasing elongation at break.

Fumed silica reduced the elongation at break by 14%. This suggests that fumed silica restricts the mobility of rubber chains. With its smaller particle size and larger surface area, fumed silica tends to form stronger interactions with the rubber matrix, limiting chain movement and thus reducing elongation. Its increased surface energy and affinity for the rubber may lead to stronger bonding, restricting the flexibility of the rubber.

Hydrated fumed silica (Sample A) improved the elongation at break by 19% compared to the untreated fumed silica. The hydration process modifies the surface characteristics of fumed silica by introducing a hydration layer, which reduces the strong interactions between silica particles and rubber chains. This results in less restriction of chain mobility and improves the rubber’s elongation and ductility.

#### 3.2.5. Resilience

The rebound resilience values of the three silica-filled vulcanized rubbers were slightly higher than those of the carbon black-filled rubber, with negligible differences among the silica samples.

#### 3.2.6. Abrasion Resistance (DIN Abrasion Teater)

The data show the impact of different fillers on the abrasion resistance of vulcanized rubber. The fillers tested included N326 carbon black, 1165MP precipitated silica, fumed silica, and Sample A (hydrated fumed silica). N326 carbon black demonstrated the best abrasion resistance, as indicated by the lowest DIN wear value, while the samples with precipitated and fumed silica displayed higher wear values.

The wear values for 1165MP precipitated silica (0.234 mm^3^) and fumed silica (0.221 mm^3^) are higher than those of carbon black, suggesting that silica fillers generally have inferior abrasion resistance. Silica’s high surface energy can lead to poor distribution in the rubber, weakening wear resistance. Fumed silica, with its large surface area, may also create weaker bonds with the rubber, making it more prone to wear.

Sample A, containing hydrated fumed silica, had a wear value of 0.21 mm^3^, slightly better than the standard fumed silica. This suggests that hydration treatment improves the abrasion resistance of silica-filled rubbers. Hydration likely enhances the filler’s dispersion and compatibility with the rubber, creating a stronger and more durable surface.

Silica fillers, including both precipitated and fumed varieties, typically have lower abrasion resistance than carbon black. Their high surface energy causes them to interact with the rubber, but this can also lead to uneven distribution, reducing wear resistance. Fumed silica, with its large surface area, may form weak bonds with the rubber matrix, making the material more vulnerable to wear.

Hydration treatment, as seen in Sample A, enhances fumed silica’s performance in rubber. This process modifies the surface properties of silica, improving its dispersion and compatibility with the rubber matrix. As a result, the rubber surface becomes stronger and more durable, leading to better abrasion resistance. Hydrated fumed silica also lowers the friction between filler particles and the rubber, further improving wear resistance.

### 3.3. Payne Effect

**Figure 9:**
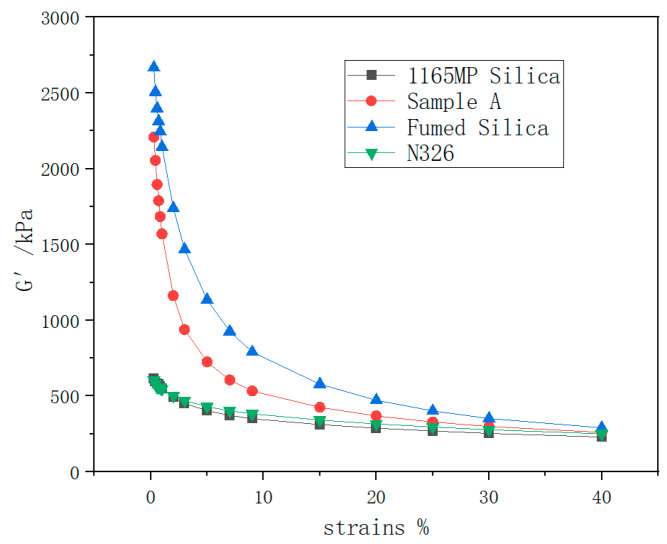
G′-strain curve of the hybrid rubber.

**Figure 10:**
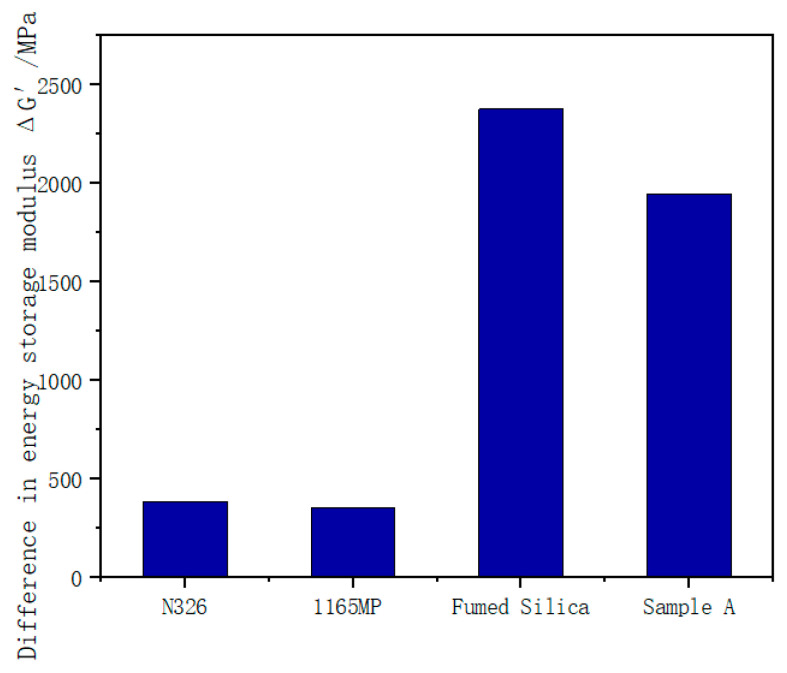
ΔG′ energy storage modulus difference in the compounds.

The relationship between the storage modulus (G′) and strain for the rubber compounds is illustrated in Figure 9, while the storage modulus difference (ΔG′) is shown in Figure 10.

#### 3.3.1. Initial Storage Modulus (G₀′)

The Payne effect is a nonlinear viscoelastic phenomenon observed in filled rubber compounds, particularly those reinforced with carbon black or silica. It describes the strain-dependent modulus reduction at low strain amplitudes in dynamic mechanical analysis. The Payne effect describes the nonlinear decrease in the storage modulus (G’) of filled rubber compounds with an increasing dynamic strain amplitude (γ\gammaγ). This effect occurs due to the progressive breakdown of the filler network structure [52].

The storage modulus G′ in the dynamic mechanical analysis is defined as follows [53]:

$$G\prime \left(\gamma \right)=\frac{\sigma_{0}}{\gamma_{0}}cos\left(\delta \right)$$

σ_0_ is the stress amplitude;

γ_0_ is the strain amplitude;

δ is the phase angle between stress and strain.

For filled rubbers, G′ decreases with an increasing strain due to the breakdown of filler–filler interactions, and this behavior is often expressed as follows:

$$\;G\;\prime \left(\gamma \right)={\;G\;\prime }_{0}-\Delta G\prime$$

G₀′ is the storage modulus at small strain (initial plateau modulus, before filler breakdown);

ΔG′ is the modulus drop due to the strain-induced breakdown of the filler network.

G₀′ represents the stiffness and strength of the filler network. A higher G₀′ indicates a denser and stiffer filler network.

The modulus drop due to strain can be empirically described as follows:

$$\Delta G\prime ={\;G\;\prime }_{0}-{\;G\;\prime }_{\infty }$$

G_∞_′ is the storage modulus at high strain, where the filler network is mostly broken down.

A common empirical model to describe this dependence is the following:

 G ′γ= G ′∞+ΔG′1+γγc mΔG′

γ_c_ is the critical strain where G′ drops significantly;

m is a fitting exponent that controls the sharpness of the modulus drop.

The modulus drop ΔG′ is directly linked to the strength of filler–filler interactions and can be influenced by filler loading (ϕ), filler dispersion, and rubber–filler interactions. A higher filler content increases G₀′ and ΔG′; poor dispersion leads to stronger agglomeration and a larger Payne effect; and stronger bonding (e.g., silane-treated silica) reduces ΔG′ [54].

Mathematically, ΔG′ is sometimes correlated with the filler volume fraction ϕ:

$$\Delta G\prime \approx k\phi^{n}$$

k and n are material-dependent constants.

Although the Payne effect is mainly described by mechanical parameters, the energy dissipation per cycle due to filler structure breakdown can be linked to thermodynamic Gibbs free energy changes:

$$\Delta G=\int_{0}^{\gamma }\;G\;\prime \left(\gamma \right)d\gamma$$

This represents the energy loss per cycle due to strain-dependent modulus reduction.

In practical applications, the higher the ΔG′, the higher the hysteresis and rolling resistance in tires, making it crucial for designing low-energy-loss rubber formulations [55].

The results in Figure 9 show that the initial storage modulus of N326 carbon black and precipitated silica compounds is relatively low, suggesting that these filler networks are less dense and exhibit lower stiffness.

In contrast, fumed silica significantly increased G₀′, indicating its ability to enhance filler network density and rigidity when used as a reinforcing filler.

Sample A (hydrated fumed silica) displayed a slightly lower G₀′ than untreated fumed silica but remained much higher than both N326 carbon black and precipitated silica. This suggests that the hydration process slightly reduces the density and rigidity of the filler network but maintains a substantial reinforcing effect.

#### 3.3.2. Storage Modulus Difference (ΔG′)

ΔG′, the difference in storage modulus between low and high strain, reflects the degree of filler network breakdown. A higher ΔG′ indicates a more easily disrupted filler network and greater dynamic energy loss in the rubber.

As shown in Figure 10:

The ΔG′ for the fumed silica compound is significantly higher than that of N326 carbon black and precipitated silica, indicating that the fumed silica filler network is more prone to breakdown under dynamic strain. This also implies the poorer dispersion of fumed silica in the rubber matrix and higher dynamic energy dissipation.

Sample A exhibited a lower ΔG′ compared to untreated fumed silica. This demonstrates that the hydration process improves the dispersion of fumed silica in the rubber matrix and strengthens the filler network, thereby reducing dynamic energy loss and enhancing the rubber’s dynamic performance.

**Figure 11:**
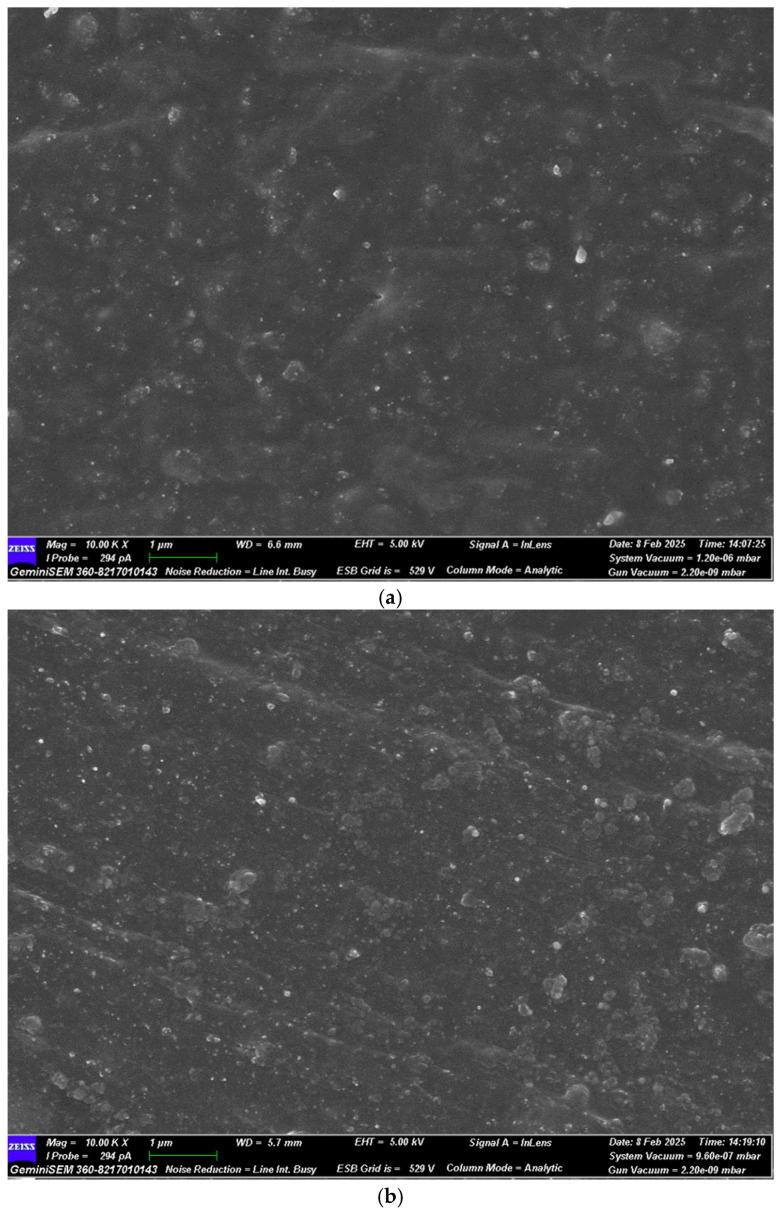

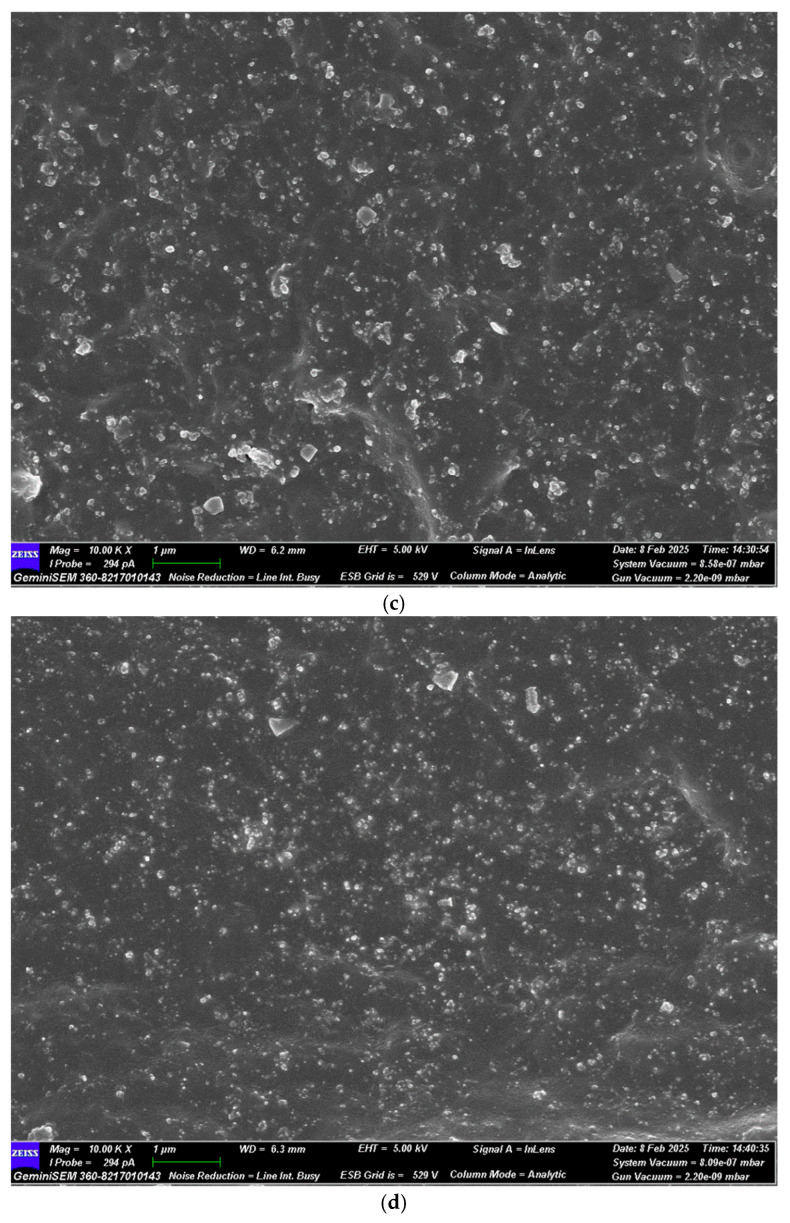
SEM micrographs of cross-sections of vulcanized rubber (10 k magnification); (**a**) N326 carbon black as the main filling material, (**b**) 1165MP silica as the main filling material, (**c**) fumed silica as the main filling material, and (**d**) Sample A as the main filling material.

As shown in Figure 11, N326 carbon black exhibits good dispersion in natural rubber, with evenly distributed particles and a strong interaction between the carbon black particles and the rubber matrix. This is due to the small particle size and large surface area of carbon black, which facilitates its effective dispersion in the rubber. Furthermore, the active surface characteristics of carbon black promote a strong interaction with the rubber, enhancing its compatibility and uniformity within the matrix.

The dispersion of 1165MP precipitated silica is relatively good, with a fairly uniform particle distribution in the SEM image. Although the particles of precipitated silica are larger and its surface energy is lower than that of carbon black, it still disperses effectively in the rubber. This suggests that the larger particle size and surface energy of precipitated silica allow for sufficient interaction with the rubber matrix.

Fumed silica exhibits poor dispersion in natural rubber, with the noticeable agglomeration of particles. The high surface area of fumed silica leads to stronger intermolecular forces between particles, resulting in agglomeration. This is because fumed silica has an extremely high surface area and surface energy, which causes the particles to attract each other and form clumps. These strong intermolecular forces hinder the proper dispersion of the filler in the rubber matrix, leading to uneven distribution.

Hydrated fumed silica (Sample A) shows slightly better dispersion than standard fumed silica, with fewer agglomerates. The SEM image shows that the hydration process improves the distribution of the filler in the rubber matrix. Hydration modifies the surface characteristics of fumed silica, enhancing its compatibility with the rubber matrix. This reduces particle attraction, improves dispersion, and minimizes agglomeration.

According to the SEM images in Figure 11, N326 carbon black and 1165MP precipitated silica exhibit good dispersion in natural rubber, while fumed silica shows poor dispersion and significant agglomeration. After undergoing hydration treatment, Sample A shows a slight improvement in dispersion. This is consistent with the results from the rubber processing analyzer, where the ΔG′ values for the vulcanizates with N326 carbon black and 1165MP precipitated silica are much lower than those of the vulcanizates with fumed silica and Sample A, with Sample A showing a significantly lower ΔG′ than fumed silica.

The higher ΔG′ values observed for the fumed silica compound indicate a greater degree of filler network breakdown under dynamic strain, which is often associated with poor dispersion and increased energy dissipation. This effect can be detrimental in industrial applications such as tire treads, where excessive hysteresis leads to higher rolling resistance and reduced fuel efficiency. The underlying mechanism for this phenomenon is the strong hydrogen bonding and high surface activity of untreated fumed silica, which promotes filler agglomeration and limits effective rubber–filler interaction.

The hydration treatment applied in Sample A mitigates these issues by improving the dispersion of fumed silica within the rubber matrix, as evidenced by the reduction in ΔG′. This suggests that hydration processing enhances the uniformity of filler distribution, leading to a more stable filler network with lower energy loss during dynamic deformation. From an industrial perspective, this improvement translates to lower rolling resistance and better fuel efficiency in tire applications. Additionally, the reduction in ΔG′ implies improved fatigue resistance and longer service life, making hydrated fumed silica a more viable alternative to traditional fillers in high-performance rubber formulations.

These findings highlight the importance of optimizing silica dispersion to balance reinforcement and dynamic properties. Future studies should further investigate the correlation between ΔG′ and real-world performance metrics such as the rolling resistance coefficient (RRC) and wet traction to validate the practical benefits of hydration processing in tread rubber applications.

### 3.4. Dynamic Mechanical Properties

**Figure 12:**
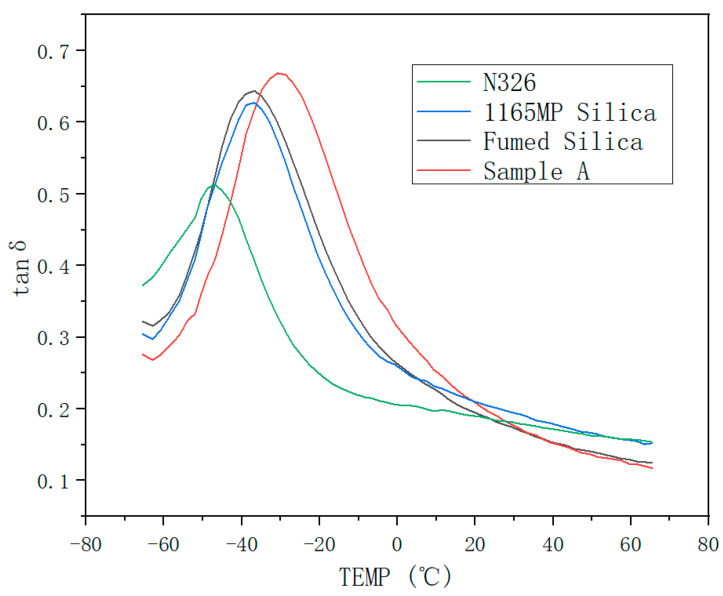
Tan δ–temperature curves of compounds.

As shown in Figure 12, compared to N326 carbon black, the damping peak of the silica-filled vulcanizates is significantly wider and higher. Among them, Sample A (hydrated fumed silica) exhibits the highest damping peak, indicating that silica fillers are more effective than carbon black in improving the damping performance of rubber. Furthermore, the hydration and drying treatment applied to fumed silica has a positive impact on enhancing the damping properties of rubber compounds.

At 0 °C, the tanδ value reflects the wet skid resistance of the vulcanized rubber; the higher the tanδ at this temperature, the better the wet skid performance.

At 60 °C, the tanδ value indicates rolling resistance; a lower tanδ at this temperature corresponds to reduced heat buildup and rolling resistance.

From Table 8, it is evident that increasing the silica content improves the wet skid resistance, as reflected by the rise in tanδ at 0 °C, while simultaneously lowering rolling resistance, as indicated by the decrease in tanδ at 60 °C. Notably, Sample A achieves the highest tanδ at 0 °C while maintaining a relatively low tanδ at 60 °C, signifying an optimized balance between grip and energy loss. This equilibrium is essential for high-performance tires, which require both enhanced traction on wet surfaces and minimized rolling resistance to improve fuel efficiency.

Hydration treatment plays a pivotal role in refining this balance by enhancing silica dispersion and reducing filler–filler interactions, leading to a more uniform energy dissipation mechanism. This improved filler network ensures that energy is effectively absorbed during deformation while preventing excessive heat buildup during rolling, which is crucial for durability and overall tire longevity. Therefore, these findings suggest that the hydration processing of fumed silica can be a viable strategy for optimizing the trade-off between damping performance and rolling resistance, making it highly applicable in next-generation high-performance tread compounds.

## 4. Conclusions

This study examined the effects of fumed silica and its hydration treatment on tread rubber performance, focusing on vulcanization characteristics, mechanical properties, the Payne effect, and dynamic mechanical behavior. The results were compared with conventional N326 carbon black and precipitated silica to assess their relative performance.

1. Vulcanization Characteristics

Fumed silica shortens vulcanization times significantly, reducing scorch time (t10) and optimum cure time (t90) by 53% and 56%, respectively, compared to N326 carbon black and precipitated silica. While this enables more efficient production, it also presents processing challenges, such as scorch control. Additionally, fumed silica exhibits the highest crosslink density (MH-ML), leading to increased strength and hardness, which enhances the durability of tread compounds.

Fumed silica and hydrated fumed silica improve hardness, tensile strength, and dynamic performance by accelerating vulcanization and increasing crosslink density. While these benefits enhance rubber durability, they also require precise process control to manage viscosity and prevent scorch. These findings underscore the potential of fumed silica-based compounds for high-performance tire applications that demand both strength and manufacturing efficiency.

2. Mechanical Properties

Fumed silica significantly enhances reinforcement, increasing 100% and 300% tensile stresses by 27% and 14%, respectively, while improving hardness by 20%.

Although fumed silica exhibited slightly lower tensile strength than N326 carbon black, hydration treatment enhanced it by 16%, making it comparable to or superior to conventional fillers.

Fumed silica reduced elongation at break by 14%, but the hydration treatment counteracted this effect, increasing elongation by 19% and improving ductility.

Fumed silica and its hydrated form improve hardness and modulus by reinforcing filler–rubber interactions and restricting molecular mobility. Precipitated silica provides moderate tensile strength improvements, while fumed silica’s rigid network slightly reduces tensile strength and elongation at break. Hydration treatment enhances dispersion, reducing stiffness and improving ductility. Although silica-based compounds show higher resilience but lower abrasion resistance than carbon black, the hydration treatment improves wear resistance by optimizing filler dispersion and interfacial bonding, making it a viable approach for enhancing tread rubber performance.

3. Payne Effect

The analysis of the Payne effect highlights the unique characteristics of the fumed silica filler network. Fumed silica exhibited a significantly higher initial storage modulus (G₀′) than N326 carbon black and precipitated silica, indicating a denser and stiffer filler network. However, it also showed a larger storage modulus difference (ΔG′), suggesting that its network is more prone to breakdown and dissipates more energy. The hydration treatment effectively reduced ΔG′, enhancing filler dispersion and improving dynamic performance.

The elevated ΔG′ values of fumed silica indicate poor dispersion and higher energy dissipation, which can negatively affect rolling resistance and fuel efficiency in tires. This behavior is primarily due to strong hydrogen bonding and filler agglomeration. The hydration treatment in Sample A improved silica dispersion, reducing ΔG′ and stabilizing the filler network. This resulted in lower energy loss, enhanced dynamic properties, and better industrial applicability. These findings suggest that optimizing silica dispersion through hydration can improve tread rubber performance by balancing reinforcement with lower rolling resistance and greater durability.

4. Dynamic Mechanical Properties

Fumed silica improved wet skid resistance, indicated by an increased tanδ at 0 °C, and reduced rolling resistance, reflected by a lower tanδ at 60 °C. The hydration treatment further optimized these properties, making it well suited for high-performance applications.

High-performance tires require an optimal balance between damping and energy dissipation. Fumed silica enhances dynamic properties such as wet skid resistance and rolling resistance while also increasing hardness and tensile modulus. However, these enhancements affect energy dissipation and damping, which are crucial for tire performance under dynamic loading.

Incorporating fumed silica in tread rubber improves both damping and energy dissipation. Its high surface area and reinforcement ability strengthen the filler network, enhancing resistance to deformation and stabilizing shear modulus across different strain levels. This ensures consistent dynamic performance under varying loads, improving grip and ride comfort. Additionally, improved dispersion reduces rolling resistance, leading to better fuel efficiency, an essential factor in high-performance tires.

The hydration and ball-milling process developed in this study enhanced the dispersion of fumed silica in the rubber matrix, ensuring more uniform energy dissipation across the tire surface. This uniformity minimizes localized wear, maintains performance consistency over time, and optimizes the tire’s dynamic response for improved handling and comfort. The combined effects of reinforcement and controlled energy dissipation significantly enhance the performance of high-performance tires, where both handling precision and durability are critical.

### Overall Findings

Fumed silica is a high-performance reinforcing filler that significantly enhances the hardness, tensile modulus, and dynamic performance of tread compounds while improving wet skid resistance and reducing rolling resistance. However, its shorter vulcanization times and higher dynamic losses may require more precise processing control. The hydration treatment of fumed silica effectively improves its dispersion and dynamic performance, making it more suitable for applications demanding a balance of mechanical and dynamic properties. These results highlight the potential of hydrated fumed silica for use in high-performance tires and other advanced rubber products.

## Figures and Tables

**Table 1:** The control group formulation.

Component	Amount (Parts)
Smoked sheet rubber (#3)	100
N326 carbon black	54
Zinc oxide	9
Stearic acid	0.4
Anti-aging agent RD	0.75
Anti-aging agent 4020	1
HDOT20	7
Accelerator DZ	0.5
Anti-scorching agent CTP	0.2

**Table 2:** The formulation for Experimental Group 1.

Component	Amount (Parts)
Smoked sheet rubber (#3)	100
1165MP silica	54
Silane coupling agent Si69	8
Zinc oxide	9
Stearic acid	0.4
Anti-aging agent RD	0.75
Anti-aging agent 4020	1
HDOT20	7
Accelerator DZ	0.5
Anti-scorching agent CTP	0.2

**Table 3:** The formulation for Experimental Group 2.

Component	Amount (Parts)
Smoked sheet rubber (#3)	100
Fumed silica	54
Silane coupling agent Si69	8
Zinc oxide	9
Stearic acid	0.4
Anti-aging agent RD	0.75
Anti-aging agent 4020	1
HDOT20	7
Accelerator DZ	0.5
Anti-scorching agent CTP	0.2

**Table 4:** The formulation for Experimental Group 3.

Component	Amount (Parts)
Smoked sheet rubber (#3)	100
Sample A	54
Silane coupling agent Si69	8
Zinc oxide	9
Stearic acid	0.4
Anti-aging agent RD	0.75
Anti-aging agent 4020	1
HDOT20	7
Accelerator DZ	0.5
Anti-scorching agent CTP	0.2

**Table 5:** Details of experimental equipment.

Instrument Name	Model	Manufacturer
Flatbed vulcanizing machine	XLD-400 × 400 × 2	Yilang Rubber Equipment Co., Qingdao, China
Mooney viscometer	PREMIER MV	American Alpha, Hudson, OH, USA
Rotorless rheometer	MDR-C	American Alpha, Hudson, OH, USA
Rubber dynamic processing analyzer	RPA-2000	American Alpha, Hudson, OH, USA
Shore durometer	WallaceH-14IRHD	Wallace & Sons, Wallingford, UK
Universal testing machine	Instron-3365	Instron Inc., Boston, MA, USA
Dynamic mechanics analyzer	GABOMETER 150	GABO, Oldenburg, Germany
Fourier transform infrared spectrometer	IS50	Thermo Fisher Scientific, Waltham, MA, USA
Demersia Fatigue Tester	FT 3000	Montec China Trading Co., Shenzhen, China
Transmission electron microscope	JEM-2100FS	JEOL, Tokyo, Japan
Planetary Ball Mills	YXQM-1L	Guru Technology, Beijing, China
Pneumatic Punching and Cutting Machine	GT7016-AR	High Speed Rail Technology Co., Beijing, China
thickness gauge	S112MXB	Mitutoyo Instruments Co., Kawasaki, Japan
DIN roller abrasion tester	SS-5643-D	China Taiwan Songjiu Testing Instrument Co., Taoyuan City, Taiwan, China
Elastomer inspection machine	SS-8350ED	China Taiwan Songjiu Testing Instrument Co., Taoyuan City, Taiwan, China
Scanning Electron Microscope	GeminiSEM 360	Carl Zeiss AG, Tübingen, Germany

**Table 6:** Mooney viscosity and vulcanization characteristics of the compounds.

	N326 Carbon Black	1165MP Silica	Fumed Silica	Sample A
ML^1+4^ (100 °C)	61.89	63.85	78.85	77.93
t_10_/min	4.01	2.62	1.87	2.15
t_90_/min	28.36	13.28	12.21	16.87
M_L_/(dN′m)	2.50	2.69	4.28	5.32
M_H_/(dN′m)	17.47	23.13	56.09	55.41
M_H_-M_L_/(dN′m)	14.93	20.44	51.81	50.09

**Table 7:** Mechanical properties of vulcanized rubber.

	N326 Carbon Black	1165MP Silica	Fumed Silica	Sample A
Hardness (Shore A)/°	65.8	67.5	80.4	78.6
100% constant tensile stress/MPa	3.14	1.87	4.27	4.24
300% constant tensile stress/MPa	14.73	10.98	17.06	16.82
Tensile strength/MPa	24.44	26.73	23.47	27.71
Elongation at break/%	428.32	578.86	375.51	463.42
Rebound value%	59.4	60.8	61.2	61.0
DIN wear value/mm^3^	0.206	0.234	0.221	0.210

**Table 8:** Moisture resistance and rolling resistance of vulcanized rubber.

	N326 Carbon Black	1165MP Silica	Fumed Silica	Sample A
Tanδ (0 °C)	0.206	0.262	0.267	0.321
Tanδ (60)	0.158	0.156	0.123	0.129

## Data Availability

Data are contained within the article.
